# Association between high-density lipoprotein-cholesterol and hypertension in relation to circulating CD34-positive cell levels

**DOI:** 10.1186/s40101-017-0143-9

**Published:** 2017-06-15

**Authors:** Yuji Shimizu, Shimpei Sato, Jun Koyamatsu, Hirotomo Yamanashi, Mako Nagayoshi, Koichiro Kadota, Shin-Ya Kawashiri, Takahiro Maeda

**Affiliations:** 1 Department of Community Medicine, Nagasaki University Graduate School of Biomedical Sciences, Nagasaki, Japan; 2 Department of Cardiovascular Disease Prevention, Osaka Center for Cancer and Cardiovascular Disease Prevention, Osaka, Japan; 3 Research and Clinical Center for Yusho and Dioxin, Kyusyu University, Fukuoka, Japan; 4 Department of Island and Community Medicine, Nagasaki University Graduate School of Biomedical Sciences, Nagasaki, Japan

**Keywords:** HDL-cholesterol, Circulating CD34-positive cell, Hypertension

## Abstract

**Background:**

Although high-density lipoprotein-cholesterol (HDL) level is inversely correlated with cardiovascular events, HDL is also reported to be positively associated with hypertension, which is a known endothelial impairment factor. Since HDL mediates important protective actions on the vascular endothelium by increasing the number of circulating endothelial progenitor cells (CD34-positive cells), the level of circulating CD34-positive cells should influence the association between HDL and hypertension.

**Methods:**

To investigate the association between HDL and hypertension in relation to the level of circulating CD34-positive cells, we conducted a cross-sectional study of 477 elderly men aged 60–69 years who participated in general health checkup.

**Results:**

HDL was found to be significantly positively associated with hypertension in subjects with a high level of circulating CD34-positive cells, while no significant association was observed for subjects with low circulating CD34-positive cells. Known cardiovascular risk factors adjusted odds (ORs) and 95% confidence intervals (CIs) of hypertension for increments of one standard deviation (SD) in HDL (13.8 mg/dL) were 1.44 (1.06, 1.96) for subjects with a high level of circulating CD34-positive cells and 0.87 (0.63, 1.19) for subjects with low circulating CD34-positive cells. We also revealed a significant association between HDL level and CD34-positive cell level on hypertension, with fully adjusted *p* values for the effect of this interaction on hypertension at 0.022.

**Conclusions:**

Independent of known cardiovascular risk factors, HDL was found to be positively associated with hypertension in subjects with a high level of circulating CD34-positive cells but not for subjects with low circulating CD34-positive cells.

## Backgrounds

A previous Japanese study reported hypertension as an important risk factor for coronary disease [1]. Additionally, low levels of high-density lipoprotein-cholesterol (HDL) are associated with increased risk of coronary artery disease [2]. These studies led us to speculate that low levels of HDL are associated with a higher prevalence of hypertension. However, a previous Japanese cross-sectional study reported that HDL is independently positively associated with hypertension in apparently healthy Japanese men and women [3].

Recently, numerous studies have reported a bidirectional association between hypertension and endothelial dysfunction—hypertension induces increased arterial stiffness and vice versa [4–10]. The cycle between hypertension and endothelial dysfunction has been established, and the process of endothelial repair should also play an important role with regard to breaking away from this vicious cycle.

On the other hand, bone marrow-derived endothelial progenitor cells such as CD34-positive cells have been reported to contribute to endothelial repair [11–13], and circulating CD34-positive cells contribute to the maintenance of cerebral circulation in an ischemic stress setting. A strong inverse correlation between the number of circulating CD34-positive cells and cerebral infarction has been shown [14]. Another study reported an inverse correlation between the level of cognitive impairment and the number of circulating CD34-positive cells in patients with vascular-type cognitive impairment [15]. Therefore, subjects with a high level of CD34-positive cells should have elevated endothelial repair activity [6, 16–18]. Since endothelial repair activity should be stimulated by endothelial injury, subjects with a high level of CD34-positive cells should present with endothelial injury similar to participants with stable cardiovascular disease and acute coronary syndrome. In addition to the above, unlike HDL from healthy subjects, HDL from participants with stable cardiovascular disease or acute coronary syndrome is reported to be lacking in endothelial anti-inflammatory effects and does not stimulate endothelial repair [19].

Given the above, we hypothesized that the positive association between HDL and hypertension occurs only in subjects with a high level of circulating CD34-positive cells.

To investigate this association, we conducted a cross-sectional study of 477 elderly men aged 60–69 years who participated in an annual health checkup from 2013 to 2015.

## Materials and methods

### Study population

This study was approved by the Ethics Committee for Human use of Nagasaki University (project registration number: 14051404). Written consent forms were available in Japanese to ensure comprehensive understanding of the study objectives, and informed consent was provided by the participants.

The study population comprised 617 male residents aged 60–69 years from the Western rural communities of Goto city and Saza town, who undertook a general medical checkup from 2013 to 2015 as recommended by the Japanese government. Persons with missing laboratory measurement data (*n =* 44) and smoking status data (*n =* 1) were excluded from the study population. To avoid the influence of inflammatory disease and hematological disease, subjects with high and low white blood cell count (≥1,000 cells/μL) (*n*=8) and 1,000 cells/μL< (*n*=2), respectively) were also excluded. Additionally, since a decline in glomerular filtration rate (GFR) was identified as a marker of chronic kidney disease (CKD), this should act as a confounding factor in the present study, and we therefore excluded 85 subjects with CKD. The remaining participants, comprising 477 men with a mean age of 65.4 years (standard deviation (SD) 2.6, range 60–69), were enrolled in the study. Hypertension, diabetes, hypercholesterolemia, and current smoker status are regarded as traditional cardiovascular risk factors [20]. Given that renal function, alcohol consumption, and hepatic enzyme levels are also important risk factors for the incidence of stroke [21, 22], and hyperuricemia is associated with carotid atherosclerosis [23], we also recognize these factors as being related to the known cardiovascular risk factors.

### Data collection and laboratory measurements

Body weight and height were measured with an automatic body composition analyzer (BF-220; Tanita, Tokyo, Japan), and body mass index (BMI; kg/m^2^) was calculated. Systolic and diastolic blood pressures were recorded at rest. Hypertension was defined as a systolic blood pressure ≥140 mmHg and/or a diastolic blood pressure ≥90 mmHg and/or taking antihypertensive medication, as described in previous studies [24, 25], since hypertension defined under these parameters was reported to be associated with the incidence of stroke and coronary artery disease in the Japanese population [1].

Fasting blood samples were collected in an EDTA-2K tube, a heparin sodium tube and a siliconized tube. The levels of white blood cells in samples from the EDTA-2K tube were measured at SRL, Inc (Tokyo, Japan).

Fresh samples (within 24 h of collection) from a heparin sodium tube were used to determine the number of CD34-positive cells. BD (Beckton Dickinson Biosciences, Franklin Lakes, New Jersey, USA) Trucount™ technology, an accurate and reproducible single platform assay conforming to the International Society of Hematotherapy and Graft Engineering (ISHAGE) guidelines [26, 27] and supported by automated software on the BD FACSCantoTM II system was used to measure the number of circulating CD34-positive cells. Serum samples were separated to measure the concentration of aspartate aminotransferase (AST) and γ-glutamyltranspeptidase (γ-GTP) using the Japanese Society Clinical Chemistry (JSCC) standardized method. Serum creatinine was measured enzymatically. HDL and low-density lipoprotein-cholesterol (LDL) were measured using a direct method, while hemoglobin A1c (HbA1c) was measured using the latex coagulation method. Serum uric acid (UA) was measured enzymatically using the uricase peroxidase (POD) method.

GFR was estimated by means of an established method that was recently proposed by the working group of the Japanese Chronic Kidney Disease Initiative [28]. According to this adapted version, GFR (mL/min/1.73 m^2^) = 194 × (serum creatinine (enzyme method))^−1.094^ × (age)^−0.287^. CKD is defined as a GFR < 60 mL/min/1.73 m^2^.

### Statistical analyses

Differences in mean values or prevalence of potential confounding factors based on circulating CD34-positive cell levels were calculated. An age-adjusted trend test was performed with a regression model for mean values, and a logistic regression model was used for proportion. Since the number of circulating CD34 cells should indicate the presence of underlying endothelial repair activity [6, 14–18] and 0.99 cells/μL was the median value of the circulating CD34-positive cells in our present study, this value was set as the cutoff point, as in our previous study [6]. Logistic regression models were used to calculate odds ratios (ORs) and 95% confidence intervals (CIs) to determine the influence of HDL on hypertension, and subjects were stratified according to the level of circulating CD34-positive cells (high, low). To avoid the influence of medication on HDL values, we also calculated ORs and 95% CIs of hypertension for increments of one standard deviation (SD) in ORs and 95% CIs stratified by circulating CD34-positive levels (high, low) limited to subjects not taking lipid-lowering medication.

Adjustments for confounding factors that we defined as known cardiovascular risk factors were made in three ways. First, we adjusted only for age. Second, we made a further adjustment for BMI based on the following: BMI has been reported to be positively associated with the level of circulating CD34-positive cells, which is divided by median values of such cells among the general population [6], and inversely associated with circulating CD34-positive cells predominantly among subjects with a high BMI (31.6 ± 5.1 kg/m^2^) [29]. In addition, unlike other cardiovascular risk factors, BMI is reported to be inversely associated with HDL-cholesterol [30] and positively associated with hypertension [31]. Therefore, BMI might act as the strongest confounding factor in the present study. Finally, for the third adjustment, we included other possible confounding factors, including Age (year) and BMI (kg/m^2^), smoking status (never smoker, former smoker, current smoker), alcohol consumption [never drinker, former drinker, current drinker (23 g/week ≤ <46 g/week, 46 g/week ≤ <69 g/week, 69 g/week≤)], HbA1C (%), LDL (mg/dL), AST (IU/L), γ-GTP (IU/L), UA (mg/dL) and GFR (mL/min/1.73 m^2^).

All statistical analyses were performed using the SAS system for Windows (version 9.4; SAS Inc., Cary, NC). All *p* values for statistical tests were two-tailed, with values of <0.05 regarded as being statistically significant.

## Results

### Characteristics of the study population

Characteristics of the present study population are shown in Table 1. Compared to subjects with a low level of CD34-positive cells, those with high levels demonstrated a significantly higher prevalence of taking antihypertensive medication as well as higher values for BMI, LDL, HbA1c, and WBC.

**Table 1:** Characteristics of study population by circulating CD34-positive cell  levels

	High CD34-positive cells (≥0.99 cells/μL)	Low CD34-positive cells (<0.99 cells/μL)	
			*p*
No. of participants	240	237	
Age, years	65.3 ± 2.7	65.6 ± 2.5	
Systolic blood pressure, mmHg	135 ± 17	134 ± 18	0.552
Diastolic blood pressure, mmHg	81 ± 11	81 ± 12	0.852
Antihypertensive medication use, %	47.1	38.4	0.031
Body mass index (BMI), kg/m^2^	23.9 ± 2.8	22.7 ± 3.0	<0.001
Current drinker, %	87.9	87.8	0.954
Current smoker, %	14.2	11.8	0.496
Serum HDL-cholesterol (HDL), mg/dL	57 ± 14	58 ± 13	0.390
Serum LDL-cholesterol (LDL), mg/dL	119 ± 29	110 ± 28	<0.001
Hemoglobin A1c (HbA1c), %	5.8 ± 0.7	5.6 ± 0.5	<0.001
Serum aspartate aminotransferase (AST), IU/L	25 ± 8	26 ± 9	0.282
Serum γ-glutamyltranspeptidase (γ-GTP), IU/L	52 ± 83	47 ± 40	0.370
Serum uric acid (UA), mg/dL	5.9 ± 1.1	5.8 ± 1.2	0.312
Glomerular filtration rate (GFR), mL/min/1.73 m^2^	76.9 ± 11.2	76.0 ± 12.9	0.926
White blood cells (WBC), cells/μL	6043 ± 1266	4993 ± 1278	<0.001

Values: mean ± standard deviation. *p* values are age-adjusted values

### Association between HDL and hypertension in total subjects and after stratification by circulating CD34-positive cell levels

Table 2 shows the ORs and 95% CIs for hypertension in relation to HDL in all subjects and as well as those in subjects stratified by circulating CD34-positive cell levels. No significant association between HDL-cholesterol and hypertension in total subjects was noted. Analysis of subjects with high levels of CD34-positive cells, however, showed that although HDL had no significant association with hypertension in an age-adjusted model, a significant positive association was observed after further adjustment for BMI. This association was unchanged even after further adjusting for known cardiovascular risk factors. Additionally, among subjects with a low level of CD34-positive cells, a tendency toward an association between HDL and hypertension was observed, although this was not significant. Even after limiting the analysis to subjects not taking lipid-lowering medication (201 men with a high level of CD34-positive cells and 210 men with low CD34-positive cells), the associations remained unchanged. Age-adjusted, age- and BMI-adjusted, and fully adjusted ORs and 95% CIs of hypertension for increments of one standard deviation (SD) in HDL (13.8 mg/dL) were 1.24 (0.95, 1.62), 1.40 (1.05, 1.86), and 1.44 (1.06, 1.96) for subjects with a high level of circulating CD34-positive cells; and 0.89 (0.68, 1.17), 1.00 (0.75, 1.33), and 0.87 (0.63, 1.19) for subjects with low circulating CD34-positive cells.

**Table 2:** Odds ratios (ORs) and 95% confidence intervals (CIs) for hypertension in relation to HDL-cholesterol stratified by circulating CD34-positive cell levels

	HDL-cholesterol tertiles			*p* for trend	1 SD increment of HDL (13.8 mg/dL)
	T1 (low)	T2	T3 (high)		
Total subjects					
No. of participants	161	155	161		
No. of cases (%)	101 (62.7)	98 (63.2)	99 (61.5)		
Age-adjusted ORs	1.00	1.02 (0.64, 1.61)	0.96 (0.61, 1.51)	0.860	1.05 (0.87, 1.27)
Age- and BMI-adjusted ORs	1.00	1.08 (0.67, 1.72)	1.22 (0.76, 1.96)	0.415	1.19 (0.97, 1.45)
Multivariable ORs	1.00	1.06 (0.66, 1.72)	1.09 (0.66, 1.79)	0.737	1.13 (0.92, 1.40)
High CD34-positive cell (≥0.99 cells/μL)					
No. of participants	89	78	73		
No. of cases (%)	53 (59.6)	52 (66.7)	51 (69.9)		
Age-adjusted ORs	1.00	1.31 (0.69, 2.48)	1.63 (0.84, 3.16)	0.142	1.24 (0.95, 1.62)
Age- and BMI-adjusted ORs	1.00	1.43 (0.75, 2.75)	2.12 (1.05, 4.27)	0.035	1.40 (1.05, 1.86)
Multivariable ORs	1.00	1.48 (0.76, 2.88)	2.34 (1.11, 4.91)	0.025	1.44 (1.06, 1.96)
Low CD34-positive cell (<0.99 cells/μL)					
No. of participants	72	77	88		
No. of cases (%)	48 (66.7)	46 (59.7)	48 (54.5)		
Age-adjusted ORs	1.00	0.77 (0.39, 1.51)	0.60 (0.31, 1.15)	0.123	0.89 (0.68, 1.17)
Age- and BMI-adjusted ORs	1.00	0.76 (0.38, 1.51)	0.72 (0.37, 1.41)	0.345	1.00 (0.75, 1.33)
Multivariable ORs	1.00	0.69 (0.33, 1.44)	0.50 (0.24, 1.05)	0.068	0.87 (0.63, 1.19)

Hypertension is defined as systolic blood pressure ≥140 mmHg, and/or diastolic blood pressure ≥90 mmHg, and/or antihypertensive medication use. Multivariable ORs: adjusted further for age and BMI, smoking status, alcohol consumption, HbA1C, LDL, AST, γ-GTP, UA and GFR. HDL tertiles are <51 mg/dL for T1, 51–61 mg/dL for T2, and >61 mg/dL for T3

### Association between HDL level and two circulating CD34-positive cell categories on hypertension

An investigation into the effects of the association between HDL level and the two CD34-positive cell categories (high and low) on hypertension revealed a significant interaction; *p* values for the effect of this interaction were *p =* 0.034 for the age-adjusted model, *p =* 0.025 for the age- and BMI-adjusted model, and *p =* 0.022 for the fully adjusted model.

## Discussion

The major findings of the present study are that, independent of known cardiovascular risk factors, HDL is significantly positively associated with hypertension among subjects with a high level of CD34-positive cells but not for subjects with low CD34-positive cells.

A previous Japanese cross-sectional study of 1803 apparently healthy men aged 49.9 ± 9.0 reported a positive association between HDL and hypertension; the adjusted OR and 95% CI of a 1 mg/dL increment of HDL for hypertension was 1.03 (1.02, 1.04, *p <* 0.001) [3]. Results of this study are compatible with our present study. Moreover, we found further evidence that the positive association is limited to subjects with a high level of CD34-positive cells.

Furthermore, in our present study, although statistical power did not reach significance initially for subjects with a high level of CD34-positive cells, after further adjusting for BMI, the association between HDL and hypertension became significant. Since HDL is significantly inversely correlated with BMI (*r =* −0.27, *p <* 0.001) and a higher BMI is a well-known independent risk factor for hypertension [31], the unfavorable influence of HDL might be masked by the favorable influence of lower BMI status.

The precise mechanism of action governing the association between HDL concentration in the blood and CD34-positive cells on hypertension risk is not understood. One plausible explanation for such an association is the inhibition of endothelial nitric oxide synthetase (eNOS) by HDL-cholesterol.

Bone marrow-derived endothelial progenitor cells such as CD34-positive cells have been reported to contribute to endothelial repair [11–13]. Endothelial cells establish an instructive vascular niche that reconstitutes hematopoietic stem and progenitor cells though the release of specific paracrine growth factors [32]. Since stimulation of CD34-positive cell production in the bone marrow is induced by damage to the endothelium, the number of circulating CD34-positive cells should increase as a result [33]. Therefore, subjects with a high level of CD34-positive cells should demonstrate elevated endothelial repair activity induced by endothelial damage. Previous studies reporting the association of limb ischemia and acute myocardial infarction with a rapid increase in circulating endothelial progenitor cells might partly support this mechanism [34, 35].

In addition to the above, unlike HDL from healthy individuals, HDL from participants with stable cardiovascular disease or acute coronary syndrome inhibits the eNOS-activating pathway and eNOS-dependent nitric oxide (NO) production that leads to loss of endothelial anti-inflammatory effects and endothelial repair [19]. Although HDL exerts vascular-protection via endothelial progenitor cells [36], our present study found a slight but significant inverse correlation between HDL and log CD34-positive cells for subjects with a high level of CD34-positive cells (*r =* −0.13, *p =* 0.047) but not for subjects with low CD34-positive cells (*r =* −0.09, *p =* 0.158).

Therefore, among subjects with a high level of CD34-positive cells, HDL values should indicate the magnitude of these unfavorable influences on endothelial repair. The number of circulating endothelial progenitor cells is reported to be inversely correlated with the risk factors for coronary artery disease [37], and the Framingham risk score [38] also should support this mechanism.

Since hypertension and endothelial dysfunction have a bidirectional association in which hypertension induces increased arterial stiffness and vice versa [4–10], an unfavorable influence on endothelial repair should cause hypertension among subjects with a high level of CD34-positive cells.

When an endothelial injury is mild, the necessity of endothelial repair is small, resulting in a low concentration of circulating CD34-positive cells. Since subjects with a small number of CD34-positive cells might have lower endothelial repair activity induced by endothelial injury, HDL should stimulate endothelial cell NO production [39] and promote endothelial repair mechanisms [40]. In subjects with a low level of CD34-positive cells, HDL should act as it does in healthy individuals. Therefore, in our present study, although the statistical power was not significant, an inverse tendency was observed between HDL and hypertension for subjects with a low level of CD34-positive cells.

Potential limitations of this study warrant consideration. Although hypertension and endothelial dysfunction have a bidirectional association, no data on the evaluation of endothelial function was available. To clarify the background mechanism of the association between HDL and hypertension in relation to circulating CD34-positive cell levels, further analysis that includes endothelial function-related data such as flow-mediated dilation (FMD) will be necessary. Although HDL demonstrates an inverse tendency with hypertension among subjects with a low level of CD34-positive cells, the statistical power did not reach significance. Further investigation with a larger sample population will be necessary. Finally, because this was a cross-sectional study, causal relationships were not able to be established.

## Conclusion

In conclusion, we found that HDL is significantly positively associated with hypertension among subjects with a high level of CD34-positive cells but not among subjects with low CD34-positive cells. These results demonstrate an efficient tool for risk estimation of HDL on cardiovascular events.

## Abbreviations

AST: Aspartate aminotransferase
BD: Beckton Dickinson
BMI: Body mass index
CI: Confidence intervals
CKD: Chronic kidney disease
eNOS: Endothelial nitric oxide synthetase
FMD: Flow-mediated dilation
GFR: Glomerular filtration rate
HbA1c: Hemoglobin A1c
HDL: High-density lipoprotein-cholesterol
ISHAGE: International Society of Hematotherapy and Graft Engineering
JSCC: Japanese Society Clinical Chemistry
LDL: Low-density lipoprotein-cholesterol
NO: Nitric oxide
OR: Odds ratio
POD: Peroxidase
SD: Standard deviation
SLS: Sodium lauryl sulfate
UA: Serum uric acid
γ-GTP: γ-Glutamyltranspeptidase
